# Leaf nutrient traits exhibit greater environmental plasticity compared to resource utilization traits along an elevational gradient

**DOI:** 10.3389/fpls.2024.1484744

**Published:** 2024-11-19

**Authors:** Xing Zhang, Jie Duan, Yuhui Ji, Weiguo Liu, Jie Gao

**Affiliations:** ^1^ Key Laboratory of Oasis Ecology of Education Ministry, College of Ecology and Environment, Xinjiang University, Urumqi, China; ^2^ Key Laboratory for the Conservation and Regulation Biology of Species in Special Environments, College of life science, Xinjiang Normal University, Urumqi, China

**Keywords:** key leaf traits, elevational gradient, climate change, soil nutrients, forest age

## Abstract

Studying key leaf functional traits is crucial for understanding plant resource utilization strategies and growth. To explore the patterns and driving factors of key leaf functional traits in forests along elevational gradients under global change, we collected survey data from 697 forests across China from 2008 to 2020. This study examined the elevational patterns of Specific Leaf Area (SLA, m²/kg), Leaf Dry Matter Content (LDMC, g/g), Leaf Nitrogen (LN, mg/g), and Leaf Phosphorus (LP, mg/g), and their responses to climate, soil nutrients, and stand factors. The results showed distinct differences in these key leaf traits at different elevational gradients. Generally, as elevation increased, SLA decreased, while LDMC significantly increased (*P* < 0.001), and LN first increase and then decreased (*P* < 0.001). The direct influence of elevation on the spatial variation of key leaf traits was greater than its indirect effects (through environmental and stand factors). The elevational patterns of leaf traits related to resource utilization strategies (SLA and LDMC) were mainly influenced by climate (temperature and precipitation) and soil nutrient factors, showing opposite trends in response to environmental changes. The patterns of leaf nutrient traits (LN and LP) along elevational gradients were primarily influenced by climatic factors, with LN exhibiting greater environmental plasticity. Compared to other stand factors, forest age predominantly influenced the spatial variation of key leaf traits, especially SLA. These findings have significant theoretical implications for revealing how plants adapt to global change.

## Introduction

Key leaf traits objectively reflect plant adaptation to environmental changes and significantly impact the structure and function of forest ecosystems (Wei et al., 2016). Common key leaf traits include Specific Leaf Area (SLA), Leaf Dry Matter Content (LDMC), Leaf Nitrogen (LN) content, and Leaf Phosphorus (LP) content (Wright et al., 2004). SLA and LDMC are commonly used to reflect trees’ resource utilization and allocation strategies (Kuppler et al., 2020; Wang et al., 2022a; Liu et al., 2023a). Leaf Nitrogen and Phosphorus contents are typically associated with trees’ growth rate and nutritional status (Tang et al., 2018). Studies have shown that the potential process of photosynthesis depends on leaf nitrogen (N) and phosphorus (P) concentrations, with trees having higher leaf N and P contents often exhibiting faster growth rates (Ellsworth et al., 2022).

Elevational gradients provide a unique natural laboratory for revealing variations in key leaf traits and their adaptations to environmental changes because they encapsulate a broad range of environmental conditions within a relatively confined geographical space (Gao and Liu, 2018). Changes in elevation are associated with systematic variations in temperature, moisture availability, and solar radiation, each of which can exert significant selective pressures on plant physiology (Shi et al., 2020; Wang et al., 2022b). Key leaf traits reflect the physiological and ecological adaptation strategies of plants under different environmental conditions (Li et al., 2021). Therefore, studying the variation patterns of key leaf traits along elevational gradients is crucial for understanding the survival and adaptation mechanisms of plants in different elevation environments (Zhang et al., 2021; Rixen et al., 2022). Moreover, research on elevational gradients can uncover how environmental factors such as climate change and soil nutrients, as well as stand factors like stand age and forest density, jointly influence key leaf traits, thereby providing a theoretical basis for predicting ecosystem functions in the context of global change. Thus, exploring the variation of key leaf traits across elevational gradients not only deepens our understanding of ecological processes but also provides strong support for species adaptive evolution and ecosystem management (Wang et al., 2022b).

Preliminary research has found that with increasing elevation, SLA significantly decreases and LDMC significantly increases, indicating a shift towards conservative survival strategies in plants (Bjorkman et al., 2018; Thomas et al., 2020). Leaf N content increases (Xu et al., 2021a), while leaf P content decreases, as low temperatures limit nitrogen mineralization rates, causing plants to accumulate more nitrogen in leaves to maintain adequate photosynthetic efficiency. Phosphorus elements are often harder for plants to absorb and utilize at high altitudes, resulting in lower leaf phosphorus content. However, some studies have observed decreases in leaf nitrogen and phosphorus contents with increasing elevation (Zhao et al., 2016; Zhang et al., 2023), suggesting that in certain ecosystems, low temperatures and soil nutrient limitations reduce the efficiency of nutrient absorption and utilization. Therefore, the patterns of key leaf traits along elevational gradients at the macro scale have not yet reached a consensus.

Along the elevational gradient, climatic factors, especially temperature and precipitation, undergo significant changes (An et al., 2020; Wang et al., 2022b). Numerous studies have found that key leaf traits are regulated by climatic factors. Under suitable temperature and precipitation conditions, trees allocate more resources to compete for light, increasing SLA and decreasing LDMC (Kuppler et al., 2020; Wang et al., 2022a; Zheng et al., 2024). Conversely, cold and arid environments increase survival stress in trees, forcing them to allocate more resources to survival. This is achieved by reducing SLA and increasing LDMC to prolong life span and slow growth, resulting in a conservative resource utilization strategy (Kramp et al., 2022; Yu et al., 2022). In cold environments, plant growth rates usually slow down, affecting their absorption and utilization of nutrients like nitrogen and phosphorus. Under drought conditions, plants may enhance nutrient acquisition by increasing root growth or altering root distribution (Wang et al., 2021a). Additionally, plants adjust the content and ratio of leaf nitrogen and phosphorus to adapt to water-limited environments (Tian et al., 2019). Furthermore, sunlight duration is another important climatic factor affecting key leaf traits. With decreasing elevation, the effective duration of sunlight reduces, leading trees to increase SLA and leaf nitrogen content and decrease LDMC. This strategy ensures efficient use of light energy and survival in intense resource competition (Reich et al., 1997).

Soil, as the direct living environment for plants, also plays a non-negligible role in key leaf traits (Wang et al., 2021b; Cui et al., 2022). Existing research shows that an increase in soil nitrogen typically leads to an increase in plant leaf nitrogen content. Higher soil nitrogen levels may cause a decrease in leaf LDMC, as plants might produce thinner, less fibrous leaves to optimize photosynthesis (Liu et al., 2021a; Pichon et al., 2022; Waring et al., 2023). Phosphorus limitation can lead to plants producing smaller leaves and higher LDMC to reduce growth demands and improve resource efficiency. Increased soil phosphorus enhances leaf phosphorus content, aiding in the synthesis of DNA and RNA and energy conversion processes, which could affect plant growth rates and leaf traits (Yang et al., 2023; Yan et al., 2024). Soil microbial activity is regulated by soil pH, and soil microbes participate in the decomposition of organic matter. Therefore, soil pH has a significant impact on plant nutrient utilization strategies and leaf nutrient traits (Kang et al., 2021; Han et al., 2023).

Forest age and stand density, among other stand factors, also have a critical impact on key leaf traits (Louis et al., 2012; Zhang et al., 2022). In the early stages of forest development, trees tend to grow rapidly, producing larger, thinner leaves (high SLA, low LDMC) to maximize photosynthesis. In the later stages of forest development, plants allocate more resources to structural reinforcement of leaves, thus increasing LDMC (Craven et al., 2018; Engbersen et al., 2022; Kramp et al., 2022). In high-density stands, competition among trees (especially for light) is more intense. This may lead to the production of smaller leaves (low SLA) and higher LDMC, adapting to lower light levels and increased competitive pressure. During the early stages of forest development, trees have a higher demand for soil nutrients, accelerating the absorption of N and P from the soil, resulting in higher leaf nitrogen and phosphorus contents (Li et al., 2017). In the later stages of forest development, the available nitrogen and phosphorus in the soil may decrease due to slower decomposition of organic matter and intensified competition between microbes and plants for nitrogen. Therefore, the leaf nitrogen and phosphorus contents in mature forests may be lower (Liu et al., 2021b, Liu et al., 2021c; Yang et al., 2021).

Based on field surveys and literature collection from 2008 to 2020, data from 697 forest sites across China were used to explore the patterns of key leaf traits along elevational gradients at a macro scale and their dominant factors. To address the above issues, we propose the following hypotheses: 1) With increasing elevation, SLA significantly decreases, and LDMC increases, indicating a more conservative plant resource utilization strategy. 2) Climatic factors are the dominant environmental factors influencing the variation of key leaf traits along elevational gradients, with stand factors (forest age) also playing a significant role. 3) The direct impact of climatic factors on the elevational variation patterns of key leaf traits is greater than their indirect effects (by affecting soil nutrient and stand factors).

## Materials and methods

### Research area and sample data

China boasts a rich variety of climate types and diverse forest ecosystems, with a forest cover of 24%. This study utilized data from 697 forests, collected through field surveys and literature between 2008 and 2020. Detailed sources are listed in **Supplementary Table S1**. 488 forest datasets from 67 sites were gathered through literature review, while the remaining 209 forest datasets from 20 sites were obtained from experiments conducted in this research. At each research site, we randomly selected at least four adjacent forest plots (30m × 30m), including typical zonal vegetation. We also recorded latitude, longitude, elevation, and slope of each site. In each plot, we mapped the spatial position of individual trees and collected the number of each tree species with a diameter at breast height (DBH) ≥ 1cm. All trees were identified by their scientific names and verified with actual herbarium specimens to confirm species identity. Forest age data was obtained through literature search and field visits, and stand density was calculated as the number of tree individuals per plot area. The forest age is primarily obtained by consulting historical records from local forestry bureaus and ecological stations, with a portion also acquired through reviewing literature sources.

### Key leaf traits

During field surveys, in each forest plot, more than 20 mature and well-developed trees (dominant trees) of each species were selected. In this experiment, we measured the fresh single leaf area of leaves without petioles using a Japanese Cano Scan LIDE 110 portable leaf area meter. The fresh leaf weight was measured with an electronic balance (precision of 0.0001 g), and the leaves were then dried in an oven at 105°C before the temperature was lowered to 60°C. After drying, the leaf dry weight was measured with a 1/10000 electronic balance. SLA (m²/kg) was calculated as leaf area/dry leaf weight, and LDMC (g/g) as leaf dry weight/fresh leaf weight. The leaf nitrogen content (LN, mg/g) was determined using the Kjeldahl method, and the leaf phosphorus content (LP, mg/g) was determined using the Mo-Sb colorimetry (Gao et al., 2023).

Due to the varying species abundance among different species, which can lead to asymmetric competition, a mathematical average trait is insufficient to represent the overall functional trait characteristics of the entire community (Cadotte, 2017; Ray et al., 2023). Therefore, we used a species abundance-based Community Weighted Mean trait (CWM) to represent the trait values of the forest.


(1) $$\text{CWM}=\sum_{\text{i}=1}^{\text{S}}{\text{D}}_{\text{i}}\;\times {\text{Trait}}_{\text{i}}$$

Here, CWM represents the community functional trait weighted characteristic value, D_i_ represents the abundance of dominant tree species, and Trait_i_ represents the selected functional trait.

### Environmental data

The study extracted mean annual temperature (MAT), mean annual precipitation (MAP), and mean annual evaporation (MAE) at a spatial resolution of 1 km from WorldClim (https://www.worldclim.org, accessed on 1 August 2023). Annual sunshine duration (ASD) is another key environmental factor influencing plant resource utilization strategies (Coble et al., 2017), and data for this were obtained from the China Meteorological Administration Meteorological Data Center (http://data.cma.cn/site/index.html, accessed on 1 August 2023). Soil pH, total soil nitrogen (N), and available soil phosphorus (P) data for the top 30 cm of soil were extracted from a 250-meter resolution grid. The soil nitrogen data is available at http://www.csdn.store (accessed on 10 April 2023), and soil available phosphorus data can be found at https://www.osgeo.cn/data/wc137 (accessed on 10 April 2023).

### Data analysis

We used Generalized Additive Models (GAMs) to explore the variation patterns of key leaf traits (SLA, LDMC, LN, and LP) at different elevational stages, where *R* ^2^ represents the model’s goodness of fit, and the *P*-value indicates the level of significant difference. This approach utilizes both parametric and non-parametric components to reduce model risks inherent to linear models (Ravindra et al., 2019). The model can be summarized as:


(2) g(E(Yi))=β0+ S1(xi)+S2(xi)+ei

where g is a link function, E(Y_i_) is the estimate for the responsible variable Y_i_, S_1_ is the smooth function of x_i_ over different treatments, S_2_ is the smooth function of x_i_ along spatial locations (Longitude and Latitude), x_i_ (i = 1, 2, 3,…, 12) are the explanatory variables, and they are number of new rhizomes, new rhizome length, new rhizome diameter, etc.β_0_ is constant term and ei is the error term. This analysis was completed using the R package “mgcv” (version 4.3.1, R Core Team, 2023).

GAMs were also used to study the contributions of various environmental factors to the spatial variation of SLA, LDMC, LN, and LP, including climatic factors (mean annual temperature [MAT], mean annual precipitation [MAP], annual mean evaporation [MAE], annual sunshine duration [ASD]), soil factors (soil nitrogen [Soil N], soil phosphorus [Soil P], soil pH), and stand factors (forest slope, forest age, tree diameter at breast height [DBH], species richness, forest density). Heatmaps were used to show correlations between different environmental factors (climate, soil, stand factors), completed using the R package “linkET”.

**Figure 1:**
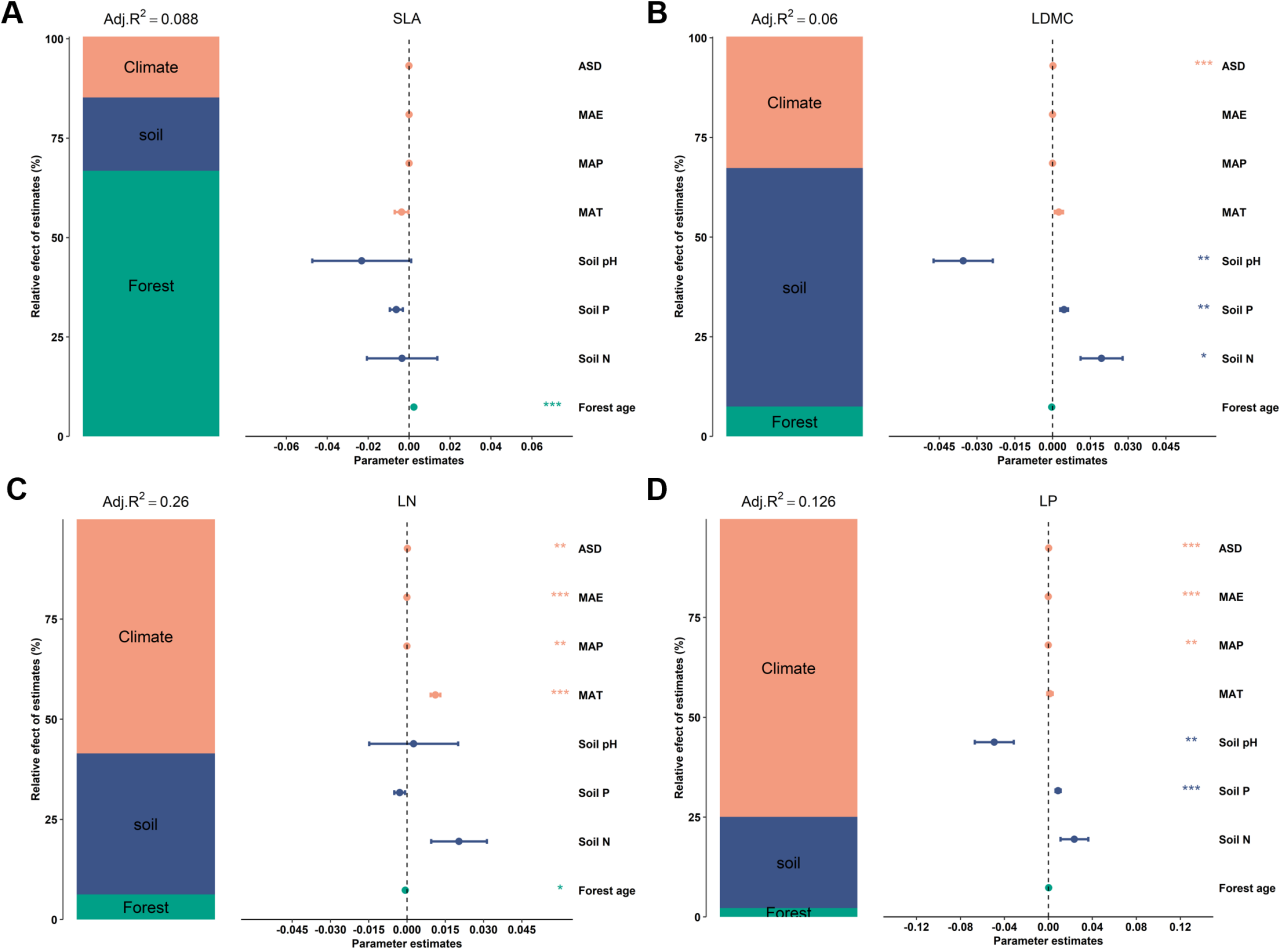
Relative effects of multiple factors on key leaf functional traits. Key leaf functional traits include: **(A)** specific leaf area (SLA); **(B)** leaf dry matter content (LDMC); **(C)** leaf nitrogen content (LN); and **(D)** leaf phosphorus content (LP). Climatic factors include: mean annual temperature (MAT); mean annual precipitation (MAP); mean annual evaporation (MAE); and annual sunshine duration (ASD). Soil factors include: soil total nitrogen content (Soil N); soil available phosphorus (Soil P); and soil pH. Stand factor includes forest age. All leaf functional trait data are log-transformed. The averaged parameter estimates (standardized regression coefficients) of the model predictors are shown with their associated 95% confidence intervals along with the relative importance of each factor, expressed as the percentage of explained variance. The adjusted (adj.) *R* ^2^ of the averaged model and the *P* value of each factor are given as: ***P < 0.001; **P < 0.01; *P < 0.05.

Variance decomposition was employed to quantify the explanatory power of climate, soil, and stand factors on the spatial variation of key forest leaf traits (**Figure 1**). This analysis was conducted in the R language package “rdacca.hp” (Lai et al., 2022). The independent contribution of each potential influencing factor to key leaf nutrient traits’ spatial variation was explored using the machine learning method of boosted regression trees, with a significance level of 0.05 for significant difference testing, performed in the R package “gbm”.

Piecewise Structural Equation Modeling (piecewiseSEM) was used to explore the impact pathways of climatic factors, soil nutrient factors, and stand factors on key leaf traits. All observed variables were initially grouped as composite variables and included in the SEM. To validate the reliability of the relationships between key ecological factors and key leaf traits, we used piecewiseSEM to elucidate the random effects of sampling points and provide “marginal” and “conditional” contributions of environmental predictors. These analyses were conducted using the “piecewiseSEM” “nlme” and “lme4” packages.

## Results

### Elevational pattern of key leaf traits

**Figure 2:**
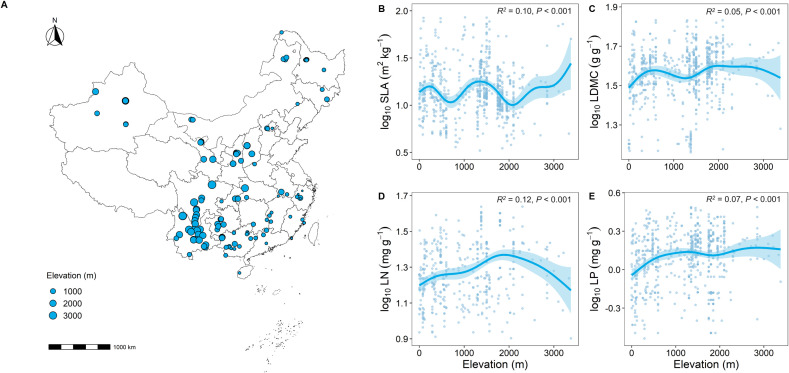
Geographic location of forest sample sites in this study and correlation analysis of key leaf functional traits with elevation. **(A)** Distribution of sample sites; **(B–E)** Relationship between key leaf functional traits and elevation. SLA represents specific leaf area, LDMC represents leaf dry matter content, LN represents leaf nitrogen content, LP represents leaf phosphorus content. All leaf functional trait data are log-transformed. *R* ^2^ represents the goodness of fit for the Generalized Additive Model, and *P*-value indicates the level of significance.

The results of the Generalized Additive Model (GAM) showed clear differences in key functional traits at different elevational gradients (**Figure 2**). Overall, as elevation increased, SLA showed a decreasing trend (**Supplementary Figure 1A**), while LDMC significantly increased (*P* < 0.001; **Supplementary Figure 1B**). Similarly, LN and LP significantly increased with elevation (*P* < 0.001; **Supplementary Figures 1C, D**).

### Climatic factors influencing key leaf traits

**Figure 3:**
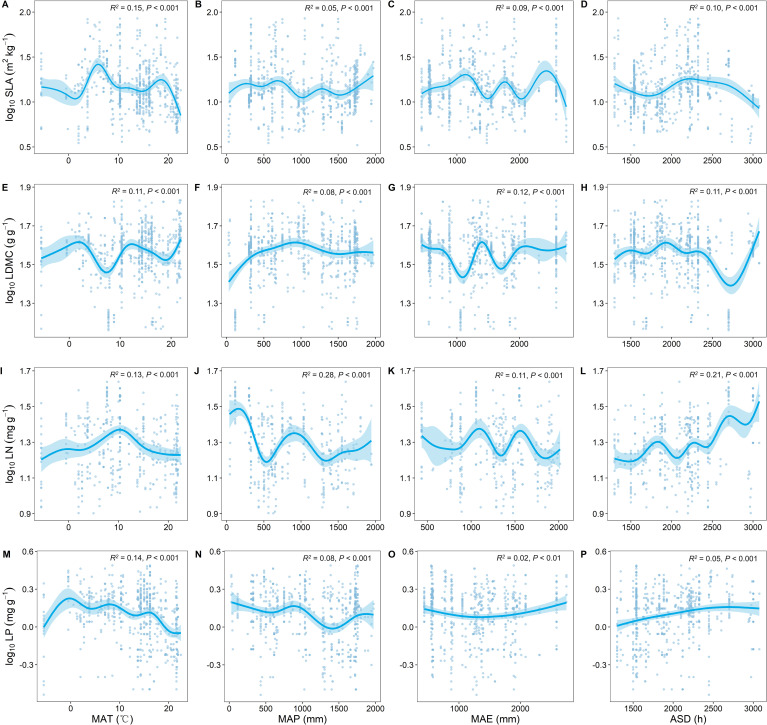
Relationship between key leaf functional traits and climatic factors. SLA, specific leaf area; LDMC, leaf dry matter content; LN, leaf nitrogen content; LP, leaf phosphorus content. Climatic factors include: mean annual temperature (MAT); mean annual precipitation (MAP); mean annual evaporation (MAE); and annual sunshine duration (ASD). All leaf functional trait data are log-transformed. *R* ^2^ represents the goodness of fit for the Generalized Additive Model, and *P*-value indicates the level of significance.

As MAT increased, SLA significantly increased and LDMC significantly decreased (*P* < 0.001; **Figure 3**). With the increase in MAT, LN showed an upward trend while LP showed a downward trend (**Figures 3I, M**). As MAP increased, both LN and LP showed a decreasing trend (**Figures 3J, N**), with LN exhibiting stronger climatic plasticity (generally higher *R* ^2^; **Figures 3I, L**).

### Soil nutrient factors influencing key leaf traits

**Figure 4:**
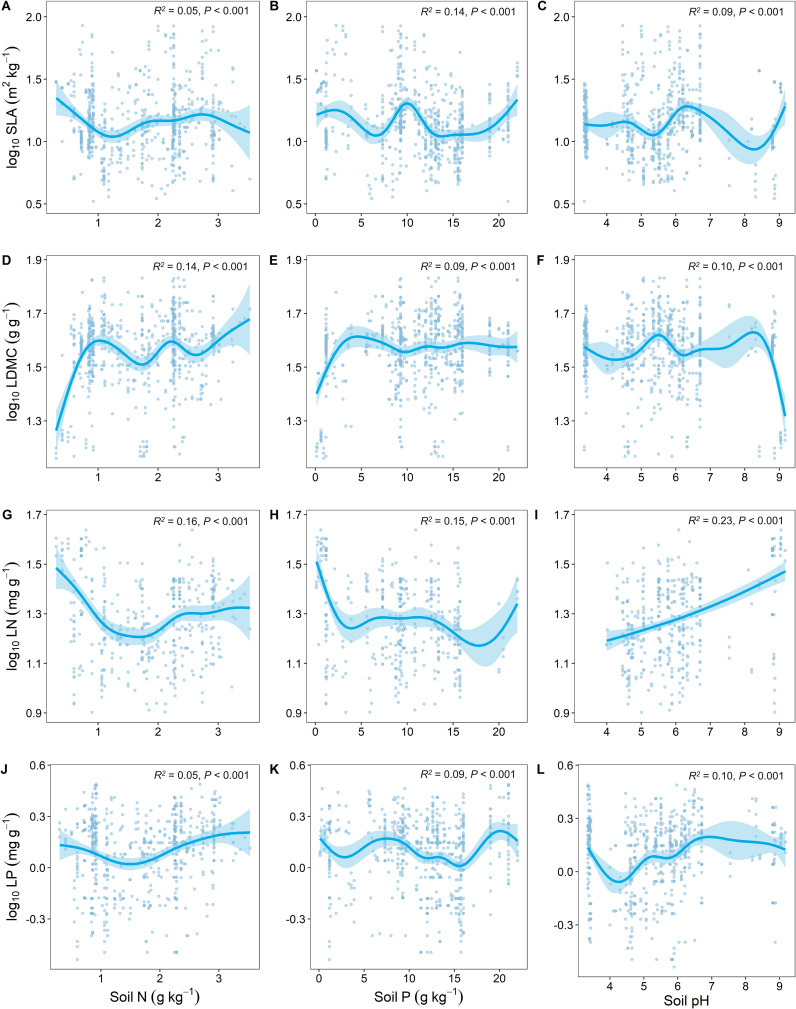
Relationship between key leaf functional traits and soil nutrient factors. SLA, specific leaf area; LDMC, leaf dry matter content; LN, leaf nitrogen content; LP, leaf phosphorus content. Soil factors include: soil total nitrogen content (Soil N); soil available phosphorus content (Soil P); and soil pH. All leaf functional trait data are log-transformed. *R* ^2^ represents the goodness of fit for the Generalized Additive Model, and *P*-value indicates the level of significance.

Soil nutrient factors (soil N, soil P, and soil pH) had the highest predictive effect on the variation of LN (**Figures 4G–I**). SLA and LDMC showed opposite trends in response to changes in soil nutrients (**Figures 4A–F**), while LN and LP showed similar trends in response to soil nutrient factors (**Figures 4G–L**).

### Stand factors influencing key leaf traits

**Figure 5:**
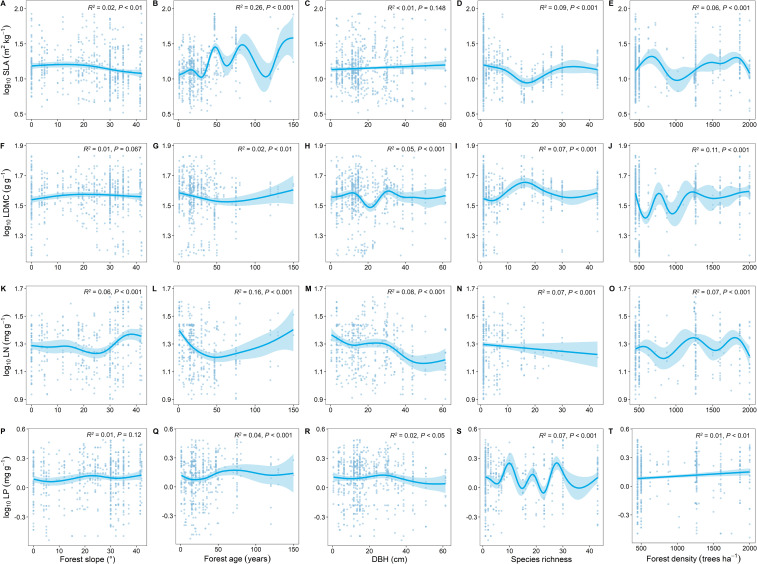
Relationship between key leaf functional traits and stand factors. SLA, specific leaf area; LDMC, leaf dry matter content; LN, leaf nitrogen content; LP, leaf phosphorus content; DBH, diameter at breast height. All leaf functional trait data are log-transformed. *R* ^2^ represents the goodness of fit for the Generalized Additive Model, and *P*-value indicates the level of significance.

Compared to other stand factors, forest age had the strongest explanatory power for the spatial variation of key leaf traits (**Figure 5**). With increasing forest age, SLA showed an increasing trend (**Figure 5B**), LDMC showed a decreasing trend (**Figure 5G**), and LN and LP showed similar trends (**Figures 5L, Q**).

### Direct and indirect effects of environmental factors on the elevational variation of key leaf traits

**Figure 6:**
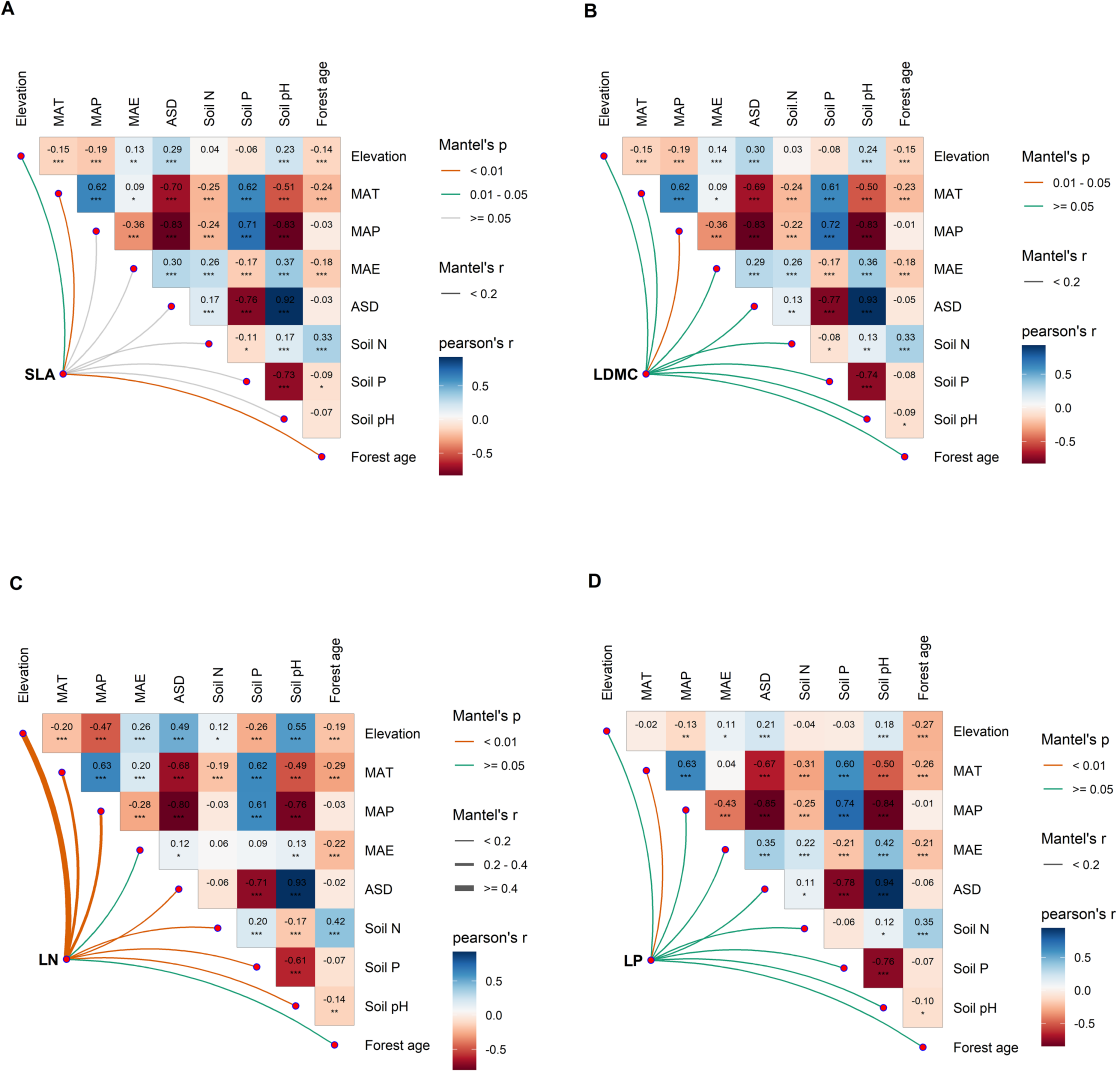
Multivariate correlation analysis of potential influencing factors of key leaf functional traits. **(A)** specific leaf area (SLA); **(B)** leaf dry matter content (LDMC); **(C)** leaf nitrogen content (LN); **(D)** leaf phosphorus content (LP). MAT, mean annual temperature; MAP, mean annual precipitation; MAE, mean annual evaporation; ASD, annual sunshine duration; Soil N, soil total nitrogen content; Soil P, soil available phosphorus content. All leaf functional trait data are log-transformed. Asterisks indicate levels of significance (***P < 0.001; **P < 0.01; *P < 0.05).

There is a significant correlation between the potential influencing factors of functional traits (**Figure 6**). Variance decomposition results indicated that leaf nutrient traits (LN and NP) have stronger (higher *R* ^2^) environmental plasticity compared to traits related to resource utilization strategies (SLA and LDMC) (**Figure 1**). Climatic factors (*R* ^2^ = 0.15, *R* ^2^ = 0.10) were the primary environmental factors influencing key leaf nutrient traits (LN and LP), though soil and stand factors also played significant roles (**Figures 1C, D**). Stand factors had the highest explanatory power for the spatial variation of SLA (*R* ^2^ = 0.059; **Figure 1A**), while soil nutrient factors were the dominant environmental factors for the spatial variation of LDMC (*R* ^2^ = 0.036; **Figure 1B**).

**Figure 7:**
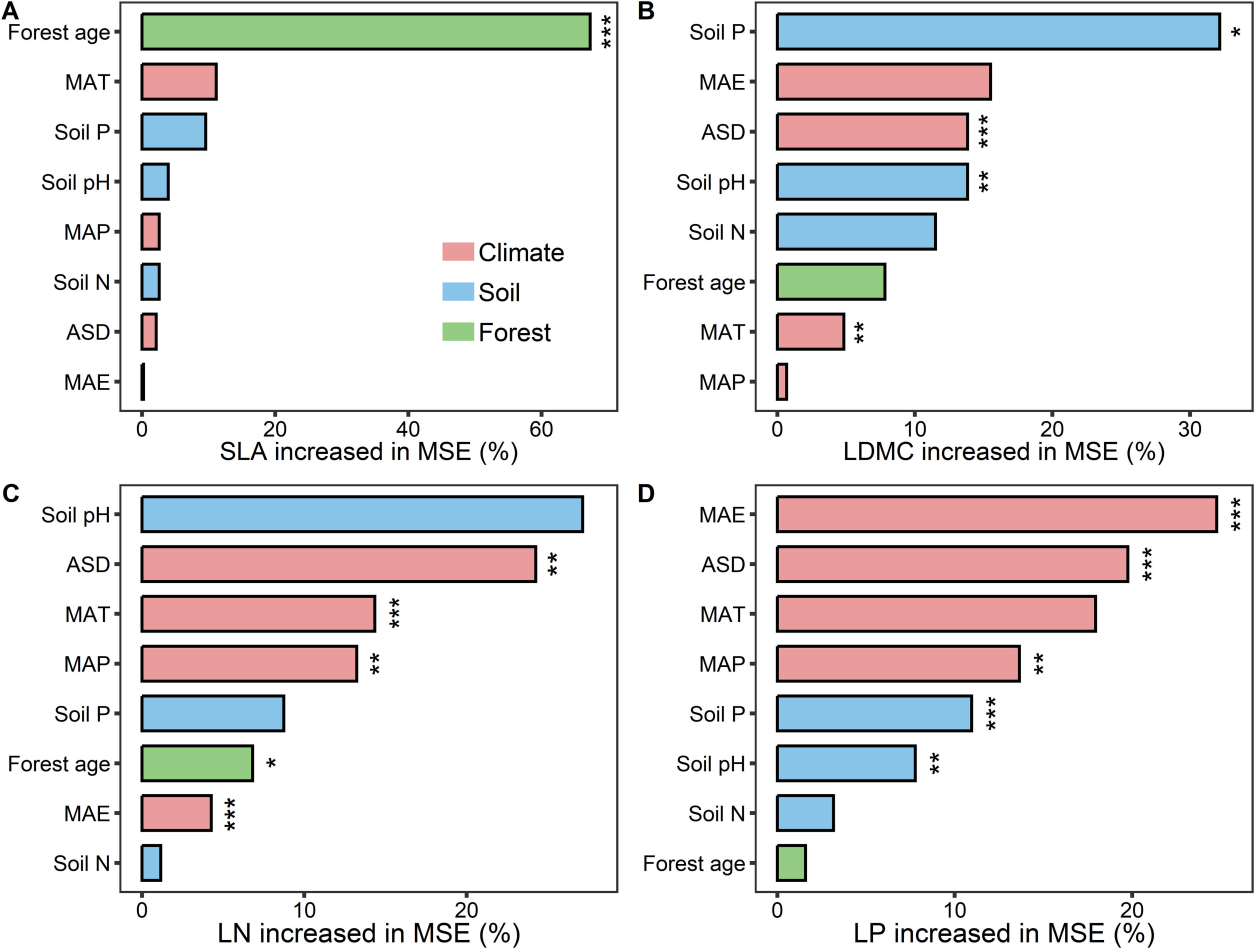
The relative importance of predictors in affecting key leaf functional traits. **(A)** specific leaf area (SLA); **(B)** leaf dry matter content (LDMC); **(C)** leaf nitrogen content (LN); **(D)** leaf phosphorus content (LP). MAT, mean annual temperature; MAP, mean annual precipitation; MAE, mean annual evaporation; ASD, annual sunshine duration; Soil N, soil total nitrogen content; Soil P, soil available phosphorus. All leaf functional trait data are log-transformed. Percentage increase in mean square error (MSE, %) of variables are used to estimate the importance of these predictors, and higher MSE% values imply more important predictors. Asterisks indicate levels of significance (***P < 0.001; **P < 0.01; *P < 0.05).

Results from the boosted regression tree model indicated that forest age had the strongest independent explanatory power for the spatial variation of SLA compared to other environmental factors (explaining 67.25%; **Figure 7A**). Soil P was the dominant environmental factor for the spatial variation of LDMC (explaining 32.1%; **Figure 7B**), and climatic factors were the main drivers for the spatial variation of LN and LP (**Figures 7C, D**).

**Figure 8:**
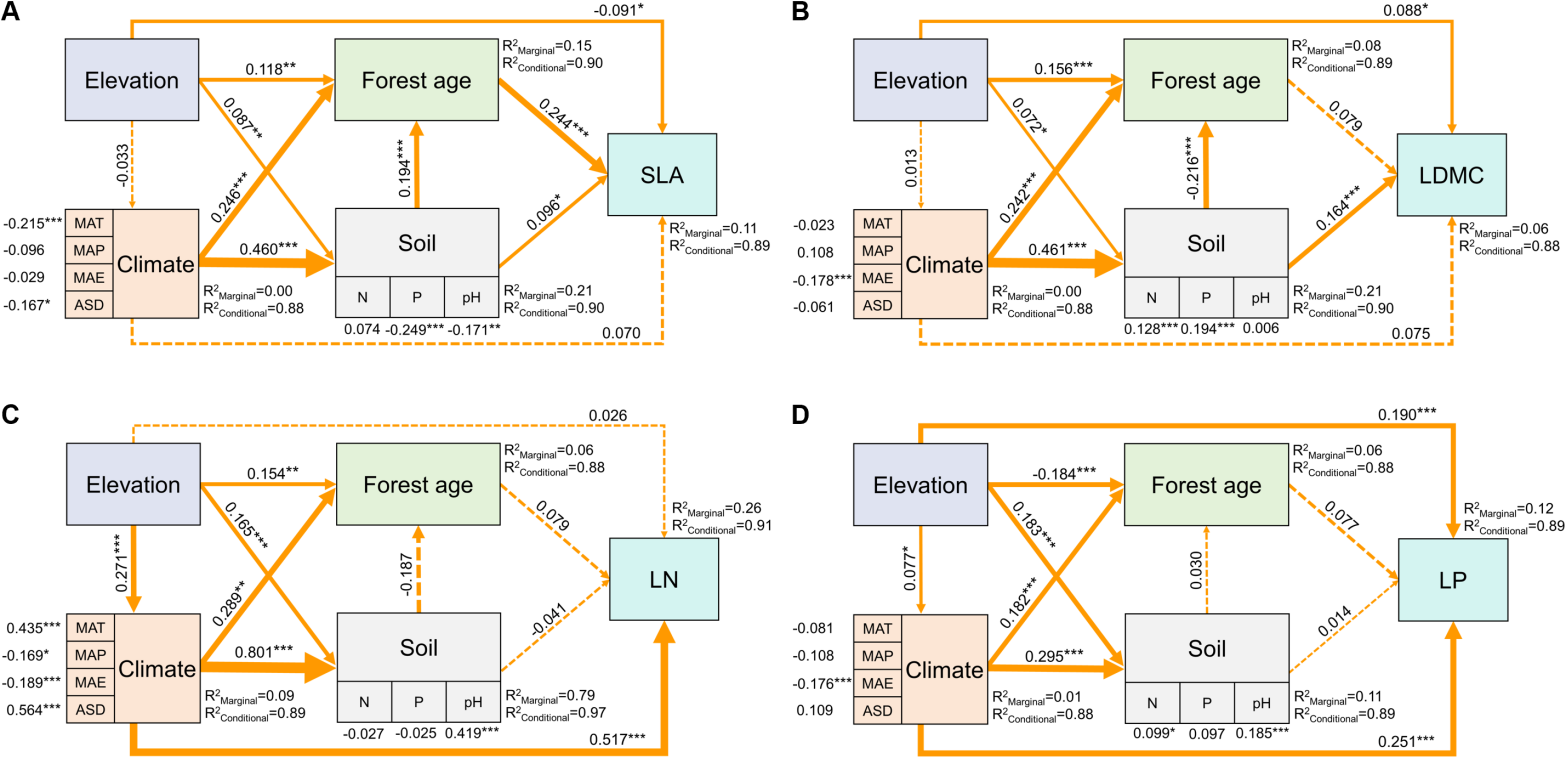
Direct and indirect driving factors affecting key leaf functional traits. Path diagrams represent the standardized results of final structural equation model (SEM) examining the relationships among variables. Numbers alongside the pathways indicate the standardized SEM coefficients, with asterisks indicating significant differences (***P < 0.001; **P < 0.01; *P < 0.05). The thickness of the arrow indicates the relative size of the path coefficient. *R* ^2^ represents the proportion of variance for each explanatory variable. All leaf functional trait data are log-transformed.

The SEM model results showed that elevation could not only directly affect SLA and LDMC but also indirectly through its impact on forest age and soil nutrients (**Figures 8A, B**). Elevation also directly influenced LN and LP, as well as indirectly through its impact on climatic factors (**Figures 8C, D**). Overall, the direct impact of elevation on the spatial variation of key leaf traits was greater than its indirect effects (**Figure 8**).

## Discussion

Trees at different elevations adopt varying survival strategies to adapt to complex habitats, often reflected in changes in key leaf traits. Our results indicate that trees in lower elevations possess higher SLA and lower LDMC, while those in higher elevations have lower SLA and higher LDMC. Rixen et al. (2022) studied intraspecific variation in aboveground functional traits across 66 alpine plant species in four countries, exploring how these traits vary with elevation. They found that SLA significantly decreases and LDMC significantly increases with increasing elevation. These findings are consistent with the results of our study. This is primarily because lower elevation areas generally have better water and thermal conditions (**Supplementary Figure 2**) (Gao and Liu, 2018), leading trees to adopt faster investment-return resource utilization strategies to cope with biotic competition in the community. These trees typically increase their light capture area, reduce leaf construction investment, resulting in higher SLA and lower LDMC (Coble et al., 2017). In contrast, higher elevation areas are generally characterized by colder temperatures, less rainfall, and fewer available soil nutrients (**Supplementary Figures 2**, **3**). Trees in these areas face survival pressure mainly from these environmental factors and adopt conservative survival strategies by reducing leaf area and increasing leaf construction investment, typically resulting in lower SLA and higher LDMC (Liu et al., 2023a). With increasing elevation, LN and LP also tend to increase. Xu et al. (2021a) sampled 428 plant individuals across 18 sampling sites along a 3000-meter elevation gradient on Gongga Mountain in China and found that leaf nitrogen content increased with elevation, consistent with the results of our study. This is mainly because the rise in elevation is usually accompanied by a decrease in temperature. Under low temperature conditions, trees’ metabolic rates decrease, leading to an increased demand for nitrogen and phosphorus to maintain normal physiological functions (Michaletz, 2018). Additionally, soils in high-altitude areas are often poor, with fewer available nutrients such as nitrogen and phosphorus. Trees adapt to these nutrient-poor environments by enhancing the efficiency of nitrogen and phosphorus absorption in roots, thereby accumulating more nitrogen and phosphorus in their leaves (Wang et al., 2021a). Our study emphasizes the significant influence of elevation on functional traits, and the observed variation in key leaf traits along the elevational gradient aligns with the leaf economics spectrum theory (Wright et al., 2004). Our findings are crucial for understanding plant adaptation, competition, ecosystem functioning, and responses to climate change across different elevations, providing valuable insights for future ecological research and forest management.

We also found that with increasing temperature, SLA significantly increases, and LDMC significantly decreases (**Figures 3A, E**). Wei et al. (2023) found that warming significantly increased SLA based on a seven-year field warming experiment, which is also consistent with our findings. This is because as temperatures rise, trees often increase their leaf area to enhance photosynthesis, thus more effectively utilizing available light. Higher SLA indicates thinner leaves, which is beneficial for light capture and gas exchange in photosynthesis (Huang et al., 2020). Increased temperatures also lead to a reduction in the accumulation of organic carbon compounds (such as cellulose and lignin) in leaves, thereby lowering LDMC. This reduction in investment in light energy allows the plant to be more efficient in growth and metabolic processes (Shi et al., 2022). As temperature increases, LN shows an increasing trend (**Figure 3I**), while with increased precipitation, both LN and LP tend to decrease (**Figures 3J, N**), which is consistent with the study findings of Chen et al. (2013). This may be because rising temperatures enhance soil microbial activity, increasing nitrogen release in the soil. Plants effectively absorb this nitrogen through their vascular systems, indirectly increasing leaf nitrogen content (Xu et al., 2021b). With continued increases in precipitation, surface runoff and subsurface flow carry away a significant amount of soil organic matter, leading to nutrient (especially nitrogen and phosphorus) depletion in the soil. Therefore, intense rainfall results in lower nitrogen and phosphorus content in plant leaves (Wang et al., 2014).

Soil, as the immediate living environment for plants, significantly influences key leaf traits. Our study found that as soil nitrogen (N) content increases, leaf nitrogen (LN) initially decreases and then increases (**Figure 4G**), which is consistent with the study findings of Chen et al. (2013). This is because when soil nitrogen content is low, plants are limited by nitrogen nutrition. They adapt by expanding their root system to seek more nitrogen sources and reduce the allocation of nitrogen in their leaves to cope with nitrogen deficiency (Wang et al., 2022c). As soil nitrogen content increases, plants can more easily absorb nitrogen. This leads to an increase in nitrogen content in leaves, enhancing photosynthesis efficiency and growth rate (Waring et al., 2023). With an increase in soil phosphorus, leaf nitrogen and phosphorus contents decrease (**Figures 4H, K**), which is contrary to the findings of Chen et al., 2013; Chen et al., 2024. According to the Nutrient Balance Theory, when a particular nutrient in the soil (e.g., phosphorus) becomes abundant, plants may alter their absorption and distribution strategies for other nutrients (e.g., nitrogen) to maintain nutrient balance (Li et al., 2023). In such cases, even if nitrogen is abundant, plants might reduce nitrogen absorption, leading to a decrease in leaf nitrogen content. The Dilution Effect also suggests that as leaf phosphorus content increases, plant growth may accelerate, causing a relative dilution of absolute nitrogen and phosphorus contents in leaves compared to the total leaf mass (Zhang et al., 2022; Liang et al., 2023). Therefore, even though the total nitrogen and phosphorus content in plants increases, the concentrations of nitrogen and phosphorus calculated per dry leaf weight might decrease. With increasing soil pH, the nitrogen and phosphorus contents in plant leaves significantly increase (**Figures 4I, L**), which is contrary to the findings of Lin et al. (2022). This discrepancy may be because acidic soil conditions can inhibit the release of soil nitrogen and phosphorus by affecting microbial activity (Liu et al., 2023b). As the pH rises, soil nitrogen and phosphorus gradually release, indirectly increasing the nitrogen and phosphorus content in plant leaves.

In addition to climatic and soil nutrient factors, stand factors, particularly forest age, significantly influence key leaf traits. As forest age increases, SLA significantly increases, LDMC decreases, and both LN and LP significantly decrease (**Figure 5**). Zhang et al. (2024) also found in their study on resource utilization strategies of trees in planted and natural forests that SLA significantly increases and LDMC significantly decreases with increasing forest age, which is consistent with our findings. As forest age changes, photosynthetic capacity and nutrient demand generally shift as trees grow. Trees at different growth stages exhibit substantial differences in physiological processes and nutrient requirements, leading to changes in resource utilization strategies and nutrient stoichiometry characteristics with increasing forest age (Zhang et al., 2022). As trees grow and mature, they may allocate more resources to the growth and maintenance of stems and roots rather than leaves (Deng et al., 2024). This means that with increasing forest age, leaf nitrogen and phosphorus accumulation may decrease to support the growth of other parts of the tree. With forest development, tree diversity increases, intensifying intra- and interspecific competition. Trees respond by increasing their light-catching surface area, typically resulting in higher SLA and reduced LDMC (Bongers et al., 2020). This competition also leads to a reduction in soil nitrogen and phosphorus content, indirectly decreasing LN and LP levels.

Leaf nutrient traits (LN and NP) exhibit greater environmental plasticity compared to traits associated with resource utilization strategies (SLA and LDMC) (**Figures 1**–**8**). In the study on the variation of plant leaf functional traits along environmental gradients and their driving factors, Akram et al. (2023) found that LN and LP are more influenced by environmental factors, particularly soil nutrients, than SLA and LDMC, which is consistent with our findings. This is primarily because they are directly related to the availability of nutrients in the environment and can quickly adjust to accommodate environmental changes (Guo et al., 2021). In contrast, SLA and LDMC, which reflect plants’ long-term adaptation strategies, change more slowly and steadily (Pierce et al., 2017). We found that stand factors, such as forest age, have the most significant direct impact on traits related to resource utilization strategies (SLA and LDMC), while climatic factors have the greatest direct influence on leaf nutrient traits (LN and LP). This is mainly because stand factors (especially forest age) directly affect traits related to resource utilization strategies by influencing plant growth strategies and resource allocation. On the other hand, climatic factors directly impact leaf nutrient traits by altering nutrient cycling and plant physiological processes, reflecting plants’ adaptability and ecological strategies under different environmental conditions (Liu et al., 2019).

Elevation not only directly affects key leaf traits but also indirectly influences them by regulating climatic, soil nutrient, and stand characteristics, with its direct impact being greater than its indirect impact (**Figure 8**). This is primarily because high-elevation environments typically present extreme physiological stress conditions, such as low temperatures, intense ultraviolet radiation, and low oxygen environments. These conditions have a direct and significant impact on plant physiological processes, forcing plants to adapt directly to survive (Abbas et al., 2022). Moreover, environmental changes caused by elevation changes (such as temperature, humidity, and light) are rapid and direct. These environmental factors directly affect plants, influencing their growth and development processes, leading to rapid adjustments in leaf traits (**Supplementary Figure 4**). Additionally, elevation’s impact on indirect factors like climate, soil nutrients, and stand characteristics is usually more complex and time-lagged (Gao and Liu, 2018). Therefore, although both direct and indirect effects of elevation influence plant leaf traits, in many cases, the direct effects, due to their immediacy, intensity, and necessity for physiological adaptation, may have a more significant impact on plants in the short term.

## Conclusions

This extensive research across various forests in China illuminates how key leaf functional traits adapt along elevational gradients, crucial for understanding plant strategies under global change. Findings reveal that with increasing elevation, SLA decreases, while LDMC, LN, and LP significantly increase. These changes are predominantly influenced by climatic and soil nutrient factors, with forest age also playing a significant role. The study highlights the substantial impact of elevation on plant functional traits, offering insights into plant adaptation strategies and guiding future ecological research and forest management.

## Data Availability

The original contributions presented in the study are included in the article/**Supplementary Material.**, further inquiries can be directed to the corresponding author/s.

## Supplementary material

Supplementary Figure 1 Relationship between key leaf functional traits and elevation. **(A)** Specific leaf area (SLA); **(B)** Leaf dry matter content (LDMC); **(C)** Leaf nitrogen content (LN); **(D)** Leaf phosphorus content (LP). All leaf functional trait data are log-transformed. *R*² represents the goodness of fit for the Linear Regression Model, and *P*-value indicates the level of significance.

Supplementary Figure 2 Relationship between climatic factors and elevation. **(A)** Mean annual temperature (MAT); **(B)** Mean annual precipitation (MAP); **(C)** Mean annual evaporation (MAE); **(D)** Annual sunshine duration (ASD). *R*² represents the goodness of fit for the Generalized Additive Model, and *P*-value indicates the level of significance.

Supplementary Figure 3 Relationship between soil nutrient factors and elevation. **(A)** Soil total nitrogen content (Soil N); **(B)** Soil available phosphorus content (Soil P); **(C)** Soil pH. *R*² represents the goodness of fit for the Generalized Additive Model, and *P*-value indicates the level of significance.

Supplementary Figure 4 Relationship between stand factors and elevation. **(A)** Forest slope; **(B)** Forest age; **(C)** Diameter at breast height (DBH); **(D)** Species richness; **(E)** Forest density. *R*² represents the goodness of fit for the Generalized Additive Model, and *P*-value indicates the level of significance.
