# Supplementary figures and images for: Leaf nutrient traits exhibit greater environmental plasticity compared to resource utilization traits along an elevational gradient
Source: Front Plant Sci. 2024 Nov 19;15:1484744. doi: 10.3389/fpls.2024.1484744 (PMC11611591; doi:10.3389/fpls.2024.1484744)

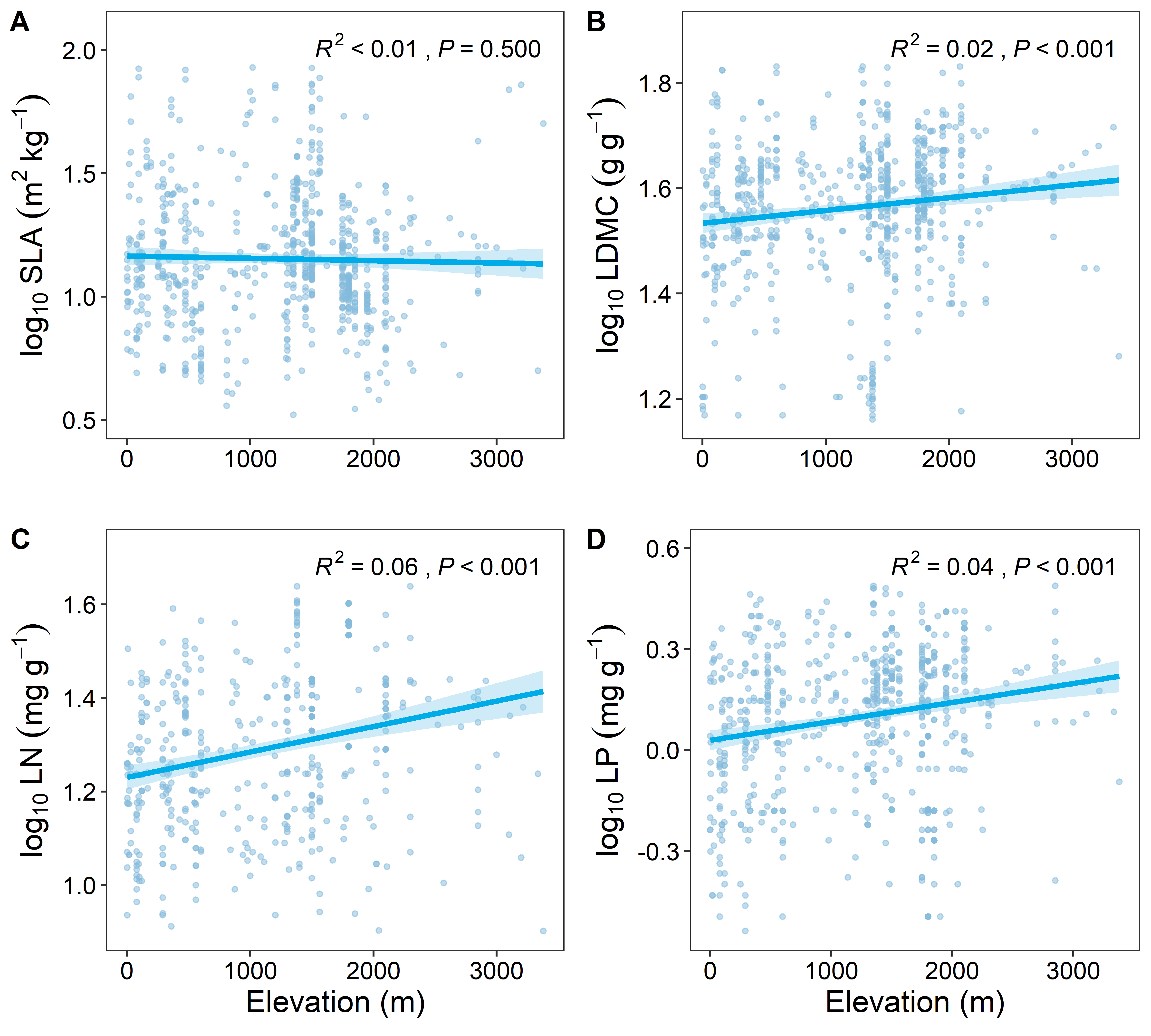

Supplement: Supplementary Figure 1 — Relationship between key leaf functional traits and elevation. (A) Specific leaf area (SLA); (B) Leaf dry matter content (LDMC); (C) Leaf nitrogen content (LN); (D) Leaf phosphorus content (LP). All leaf functional trait data are log-transformed. R² represents the goodness of fit for the Linear Regression Model, and P-value indicates the level of significance. [file Presentation1.zip › Supplementary Figures/Supplementary Figure 1.tif]

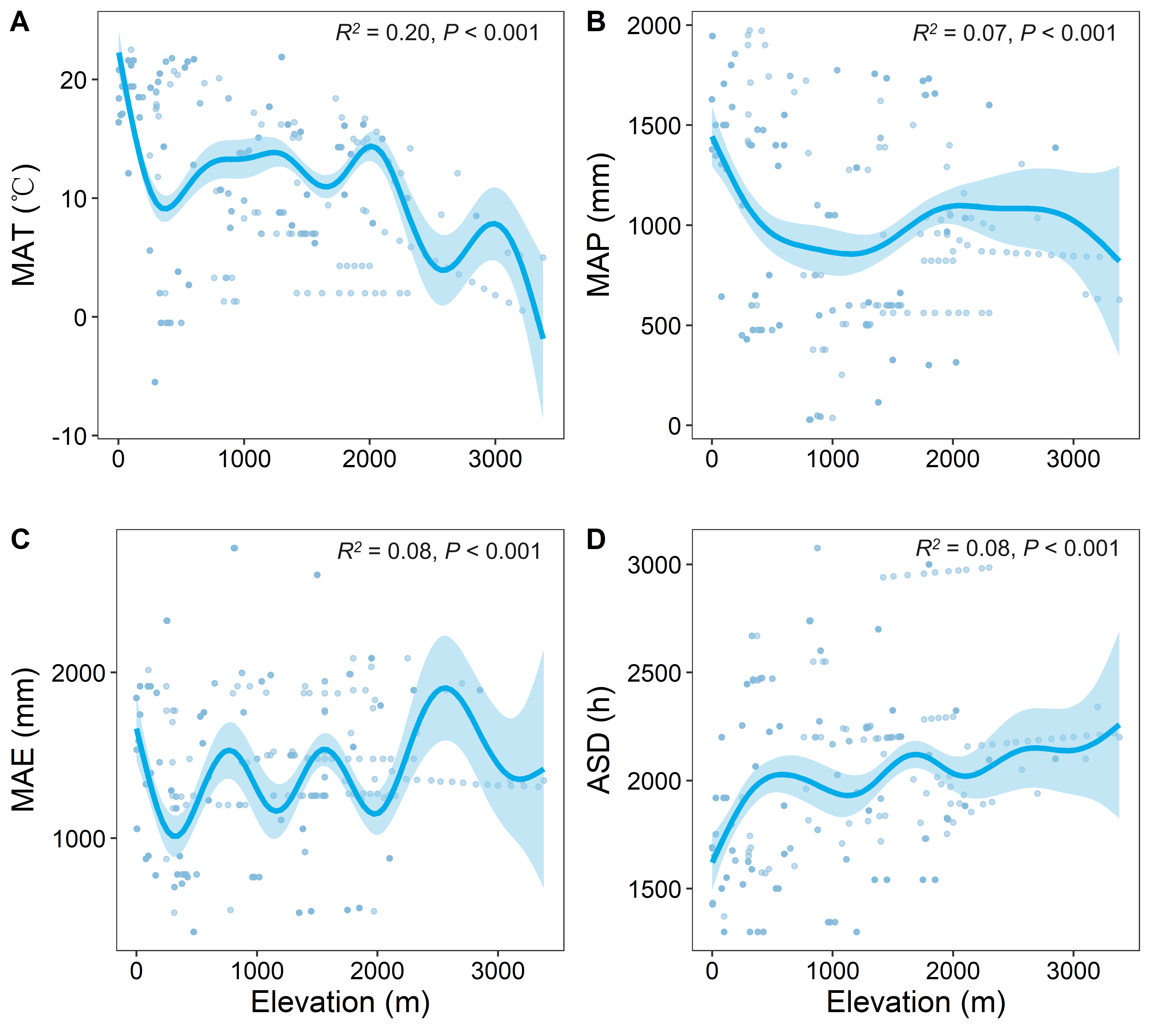

Supplement: Supplementary Figure 1 — Relationship between key leaf functional traits and elevation. (A) Specific leaf area (SLA); (B) Leaf dry matter content (LDMC); (C) Leaf nitrogen content (LN); (D) Leaf phosphorus content (LP). All leaf functional trait data are log-transformed. R² represents the goodness of fit for the Linear Regression Model, and P-value indicates the level of significance. [file Presentation1.zip › Supplementary Figures/Supplementary Figure 2.tif]

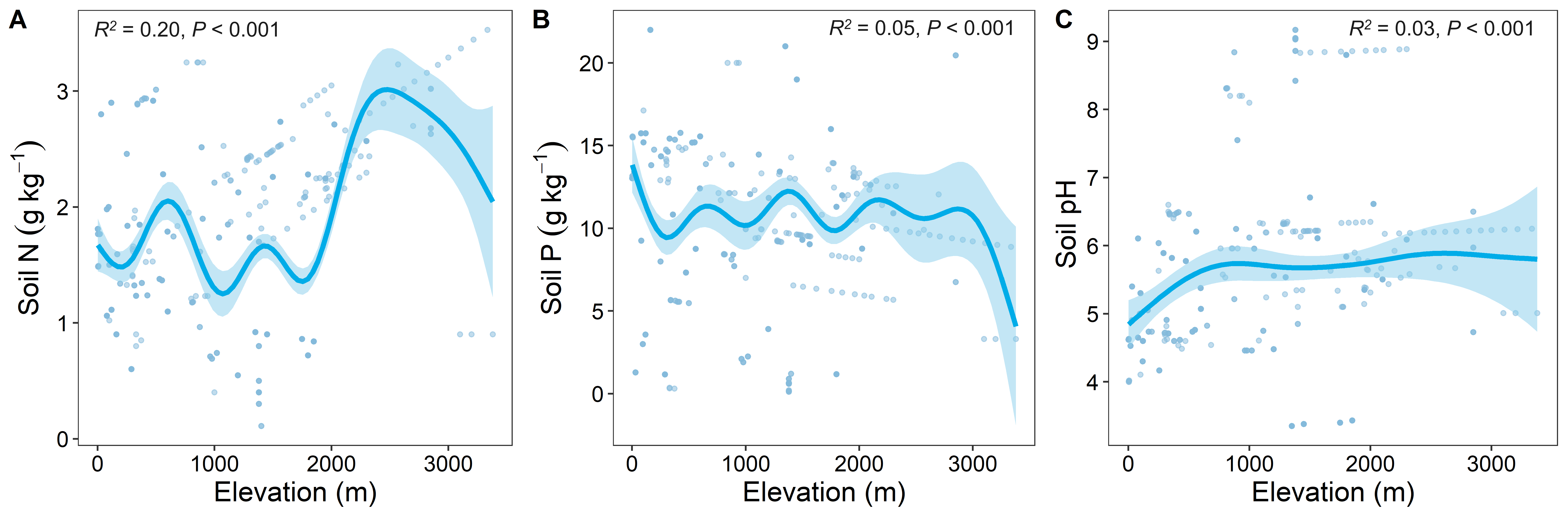

Supplement: Supplementary Figure 1 — Relationship between key leaf functional traits and elevation. (A) Specific leaf area (SLA); (B) Leaf dry matter content (LDMC); (C) Leaf nitrogen content (LN); (D) Leaf phosphorus content (LP). All leaf functional trait data are log-transformed. R² represents the goodness of fit for the Linear Regression Model, and P-value indicates the level of significance. [file Presentation1.zip › Supplementary Figures/Supplementary Figure 3.tif]

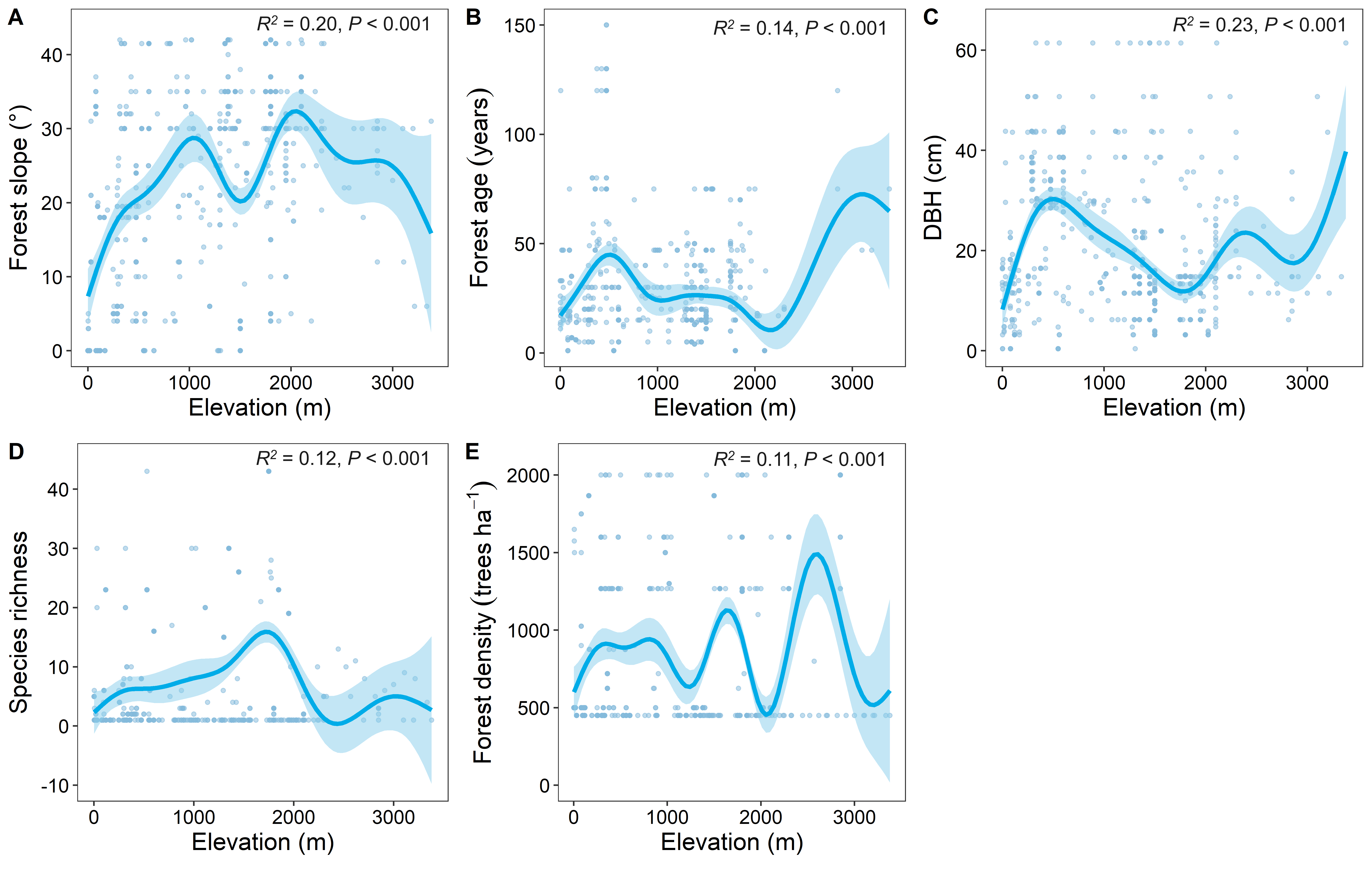

Supplement: Supplementary Figure 1 — Relationship between key leaf functional traits and elevation. (A) Specific leaf area (SLA); (B) Leaf dry matter content (LDMC); (C) Leaf nitrogen content (LN); (D) Leaf phosphorus content (LP). All leaf functional trait data are log-transformed. R² represents the goodness of fit for the Linear Regression Model, and P-value indicates the level of significance. [file Presentation1.zip › Supplementary Figures/Supplementary Figure 4.tif]
